# *Bacteroides fragilis* and propionate synergize with low-dose methimazole to treat Graves’ disease

**DOI:** 10.1128/spectrum.03186-24

**Published:** 2025-04-23

**Authors:** Xinjie Zhang, Qingxin Pan, Guixiang Yao, Danxia Kong, Haiyan Chen, Qunye Zhang, Zhe Wang

**Affiliations:** 1 Department of Geriatrics, Department of Endocrinology, Shandong Provincial Hospital Affiliated to Shandong First Medical University, Jinan, Shandong, China; 2 Department of Biology, University College London 98552, London, United Kingdom; 3 Shandong First Medical University Second Affiliated Hospital 573752 , Tai'an, Shandong, China; 4 Key Laboratory of Cardiovascular Remodeling and Function Research, Chinese Ministry of Education, Chinese National Health Commission and Chinese Academy of Medical Sciences, Jinan, Shandong, China; 5 Department of Cardiology, Qilu Hospital of Shandong University 162759, Jinan, Shandong, China; 6 State Key Laboratory for Innovation and Transformation of Luobing Theory, Jinan, Shandong, China; 7 Department of Occupational Lung Disease, Shandong Academy of Occupational Health and Occupational Medicine, Jinan, Shandong, China; Lerner Research Institute, Cleveland, Ohio, USA

**Keywords:** Graves' disease, *Bacteroides fragilis*, propionate, autoimmunity, gut microbiota-based treatments

## Abstract

Graves’ disease (GD) is a common autoimmune thyroid disease with limited treatment efficacy, high relapse rates, and severe adverse effects. Gut microbiota dysbiosis is thought to play a critical role in GD, with the potential for microbiota-based therapies in GD treatment. However, experimental evidence is needed to validate this hypothesis. In this study, we evaluated the therapeutic effects of the commensal bacterium *B. fragilis* and its metabolite, propionate, in a GD mouse model. We found that oral supplementation with *B. fragilis* or propionate significantly reduced serum levels of inflammatory cytokines, total thyroxine (TT4), and TSH receptor antibodies (TRAb). It also decreased the proportion of circulating Th17 cells while increasing the proportion of circulating regulatory T cells (Tregs), thus mitigating systemic inflammation, hyperthyroidism, and the autoimmune response against TSHR. In addition, *B. fragilis* and propionate significantly decreased the levels of inflammatory cytokines and the proportion of M1 macrophages in thyroid tissue, while increasing the proportions of Treg cells and M2 macrophages, thereby reducing thyroid inflammation and size. Notably, the combination of *B. fragilis* or propionate with methimazole (MMI) significantly ameliorated pathological changes in GD mice and markedly reduced MMI dosage requirements, demonstrating a synergistic therapeutic effect. Our findings suggest that *B. fragilis* and propionate could serve as effective adjuvant agents in combination with MMI, thereby enhancing therapeutic efficacy while reducing drug dosage and minimizing side effects. This study opens a new avenue for microbiota-based treatments in managing GD.

**IMPORTANCE:**

This study explores a new approach to treat Graves’ disease (GD), a major type of hyperthyroidism. Traditional treatments for GD often come with significant side effects and high relapse rates. Researchers found that *Bacteroides fragilis*, a gut commensal bacterium, and its metabolite propionate can improve the condition of GD mice. When combined with methimazole, a conventional medication for GD treatment, these natural agents demonstrated enhanced therapeutic efficacy, enabling dose reduction of methimazole and consequently reducing adverse effects. This research suggests that combining gut microbiota-based treatments with standard therapies may offer a more effective and safer way to manage GD.

## INTRODUCTION

Graves’ disease (GD) is a prevalent thyroid-specific autoimmune disease and the leading cause of hyperthyroidism (1). GD development is primarily driven by thyroid-stimulating antibodies (TSAb) that target the thyroid-stimulating hormone receptor (TSHR) and mimic the effect of thyroid-stimulating hormone (TSH), leading to excessive proliferation of thyroid follicular cells and the overproduction of thyroid hormones (2). The pathogenesis of GD involves a complex interplay of genetic (such as CTLA-4, PTPN22, and HLA) and environmental factors (including infections, stress, and iodine intake), which collectively contribute to autoimmune dysregulation and the generation of antibodies against thyroid tissues (3). GD poses significant health risks, including hypermetabolism, cardiovascular disorders, Graves’ ophthalmopathy, and pretibial myxedema. Severe cases can lead to life-threatening complications, such as heart failure and thyroid storm.

Current treatments of Graves’ disease (GD) primarily include antithyroid drugs (ATDs, such as methimazole), radioactive iodine therapy, and thyroidectomy (4). However, ATDs have drawbacks including prolonged usage, high relapse rate (40%–50%), and adverse effects such as agranulocytosis, liver dysfunction, and fetal malformations (5, 6). ^131^I therapy, although effective in mitigating hyperthyroidism, often leads to permanent hypothyroidism, necessitating lifelong thyroid hormone replacement, and may exacerbate Graves’ ophthalmopathy (7). Thyroidectomy, while providing rapid relief from hyperthyroidism, carries inherent risks, such as hypothyroidism, hypoparathyroidism, and recurrent laryngeal nerve injury (8). Given these limitations, it is imperative to further investigate the pathogenesis of GD and optimize existing treatment regimens.

The human gut harbors approximately 10¹⁴ bacteria, forming a complex microecosystem that plays a crucial role in maintaining human health (9–11). Recent studies have identified gut microbiota dysbiosis as a significant etiological factor for various autoimmune diseases, including rheumatoid arthritis, multiple sclerosis, and ankylosing spondylitis (12–16). Gut dysbiosis can disrupt the intestinal barrier, allowing pathogens and toxins (e.g., LPS) to enter the circulation and trigger systemic inflammation. It also affects the production of microbial metabolites, such as short-chain fatty acids (SCFAs), affecting immune cell function and consequently initiating autoimmune responses. Gut microbiota can modulate T-cell differentiation and function, including regulatory T cells (Tregs) and helper T cells (Th), through metabolites such as SCFAs and bile acids (3, 17, 18). Our previous research showed that GD patients exhibit significant gut dysbiosis, characterized by a reduced abundance of SCFA-producing bacteria like *Bacteroides fragilis* (BF) and decreased SCFA levels in the gut and serum. Moreover, these abnormalities are closely related to disease severity, immune dysregulation, and a reduced Treg/Th17 ratio in GD patients (19). These findings highlight gut dysbiosis as a critical pathogenic mechanism in GD, presenting a potential novel therapeutic target.

*B. fragilis*, a common gut commensal bacterium, can ferment polysaccharides to produce SCFAs, such as propionate and butyrate, which are essential for maintaining gut barrier function, regulating immune responses, and inhibiting pathogen growth. SCFAs have been reported to modulate NLRP3-mediated inflammation by suppressing macrophage activation and the release of IL-18 and IL-1β (20). Our previous research demonstrated that the abundance of gut *B. fragilis* is significantly reduced in GD patients, which affects Treg and Th17 cell differentiation through propionate (19). Numerous studies have shown that propionate maintains intestinal barrier integrity and immune system balance and inhibits excessive inflammation and autoimmune responses. Decreased propionate levels in the gut and circulation are closely associated with various autoimmune diseases, such as multiple sclerosis and rheumatoid arthritis. Recent studies demonstrate that gut microbiota-based therapies, including probiotics, prebiotics, and fecal microbiota transplantation (FMT), exhibit significant potential in the treatment of autoimmune diseases (21–23). Therefore, *B. fragilis* and propionate warrant investigation as potential intervention targets for maintaining immune tolerance and treating autoimmune diseases.

Herein, we evaluate the therapeutic effects of *B. fragilis* and propionate, both as standalone treatments and in combination with MMI using a GD mouse model. We found that these interventions could attenuate systemic and local inflammation and enhance the therapeutic efficacy of MMI while minimizing its dosage. Our findings suggest that *B. fragilis* and propionate could serve as adjuvant therapies for GD.

## RESULTS

### Gut microbiota dysbiosis exacerbates disease severity in GD mice

**Fig 1:**
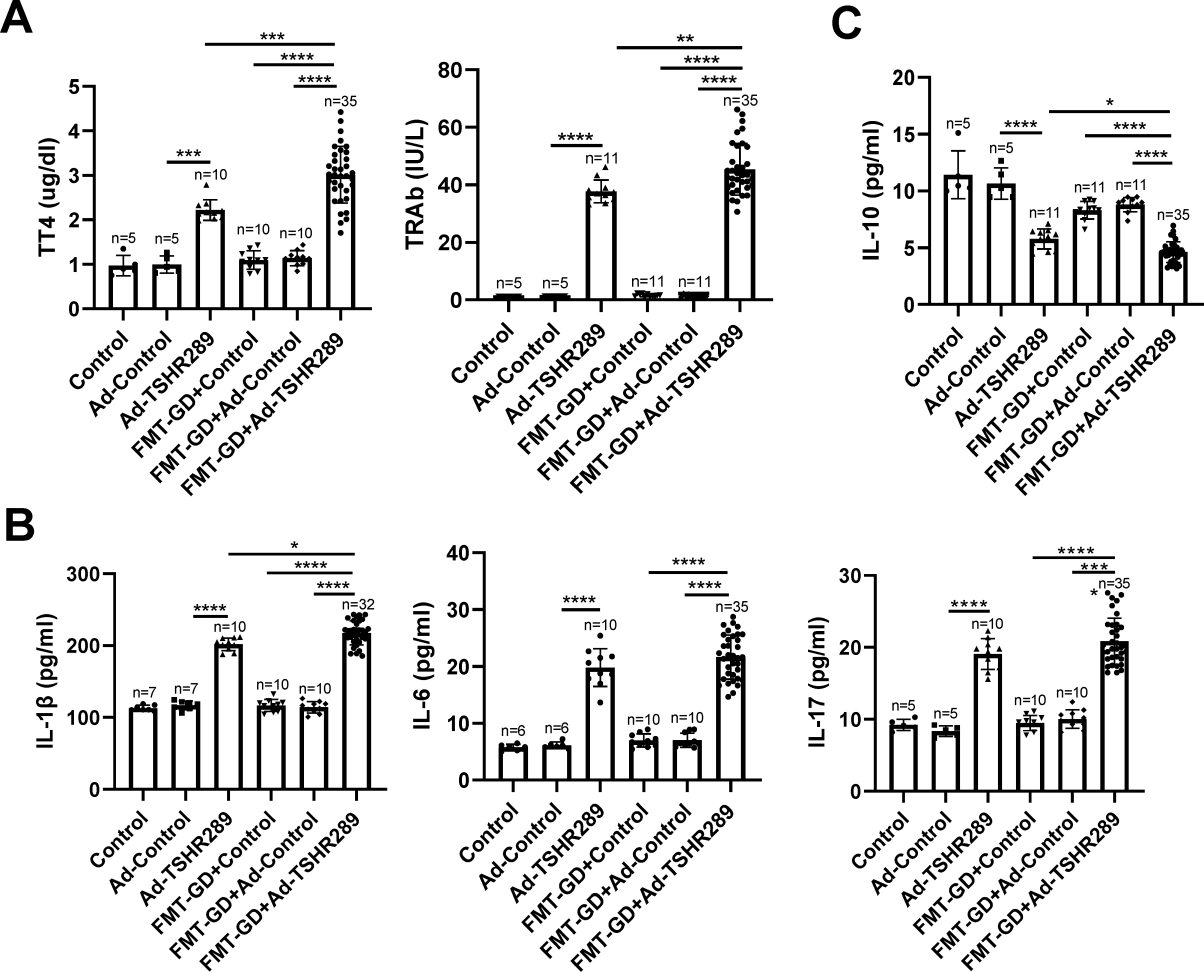
Serum levels of TT4, TRAb, and inflammatory cytokines in mice. (**A**) Comparison of serum TT4 (left panel) and TRAb (right panel) levels among different groups of mice. (**B and C**) Comparison of serum levels of pro-inflammatory cytokines IL-1β, IL-6, and IL-17 (**B**) and the anti-inflammatory cytokine IL-10 (**C**) among different groups of mice. The sample size for each group is indicated in the figure. Control: mice treated with PBS; Ad-Control: mice intramuscularly injected with an empty adenovirus vector. Ad-TSHR289: mice intramuscularly injected with adenovirus vector Ad-TSHR289. FMT-GD + Control: Control group mice transplanted with fecal microbiota from GD patients. FMT-GD + Ad-Control: Ad-Control group mice transplanted with fecal microbiota from GD patients. FMT-GD + Ad-TSHR289: Ad-TSHR289 group mice transplanted with fecal microbiota from GD patients. Statistical analysis was performed using one-way ANOVA followed by the Tukey-HSD test. *P < 0.05; **P < 0.01; ***P < 0.001; ****P < 0.0001.

To evaluate the therapeutic effects of *B. fragilis* and propionate in GD, we established a GD mouse model by immunizing BALB/c mice with an adenoviral vector expressing amino acid residues 1–289 of the TSHR (Ad-TSHR289) (Fig. S1A). Infection experiments using 293T cells confirmed the high infectivity of the adenovirus vector (Fig. S1B and C). Based on our previous findings (19), we transplanted fecal microbiota from GD patients to the GD model mice to enhance the success rate of model construction (Fig. S1A). After FMT, the gut microbiota of recipient mice exhibited a high degree of overlap with that of GD patients (donors), demonstrating the successful FMT (Fig. S1D). Compared to untreated mice (Control group) and mice infected with an empty adenoviral vector (Ad-Control group), GD model mice infected with Ad-TSHR289 (Ad-TSHR289 group) exhibited significantly increased levels of TT4, TRAb, and pro-inflammatory cytokines (IL-1β, IL-6, and IL-17), with a significant reduction in anti-inflammatory IL-10 levels (Fig. 1A through C). The transplantation of gut microbiota from GD patients did not significantly affect thyroid function (serum TT4 and TRAb levels) or serum inflammatory cytokine levels in euthyroid control mice (FMT-GD + Control and FMT-GD + Ad-Control vs. Control and Ad-Control, respectively). However, compared to GD mice (Ad-TSHR289 group), transplantation of gut microbiota from GD patients significantly exacerbated hyperthyroidism, TSHR autoimmunity, and systemic inflammation (FMT-GD + Ad-TSHR289 group) (Fig. 1A through C). These results suggest that gut microbiota dysbiosis alone may not be sufficient to induce GD, but can enhance the effects of pathogenic factors like Ad-TSHR289, thereby promoting the onset and progression of GD.

### Oral supplementation with *B. fragilis* or propionate significantly increases their levels in the gut and/or circulation of GD mice

**Fig 2:**
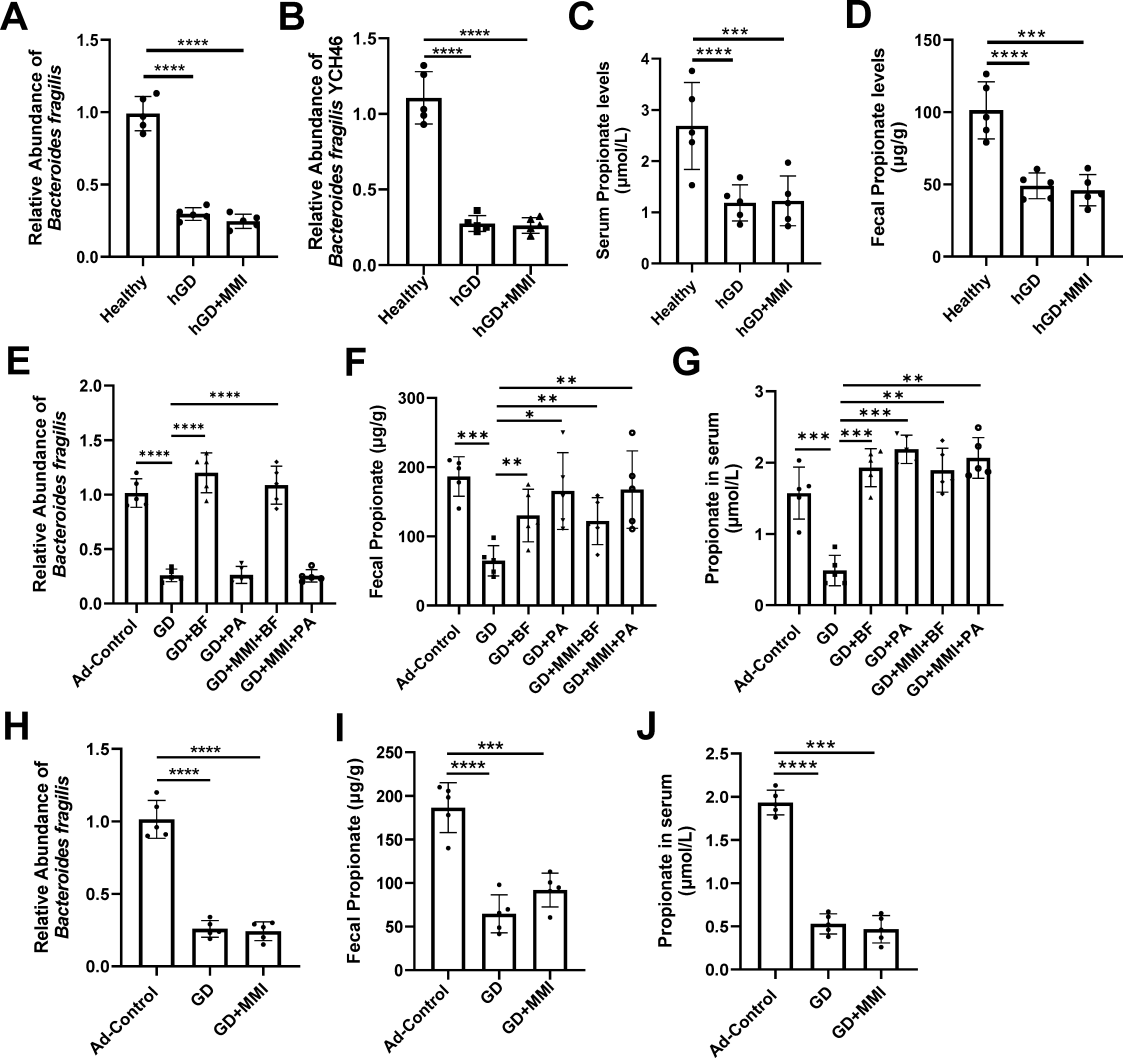
Abundance of *B. fragilis* and propionate in the intestine and/or serum of GD patients and mice. (**A and B**) Comparison of the intestinal abundance of *B. fragilis* (**A**) and *B. fragilis YCH46* (**B**) among different groups of GD patients. *n* = 5. (**C and D**) Comparison of propionic acid levels in serum (**C**) and intestine (**D**) among different groups of GD patients. *n* = 5. Healthy: healthy individuals; hGD: GD patients; hGD + MMI: GD patients treated with MMI. (**E and F**) Comparison of intestinal abundance of *B. fragilis* (**E**) and propionate (**F**) among different groups of mice, *n* = 5. (**G**) Comparison of serum propionate levels among different groups of mice, *n* = 5. Ad-Control: mice intramuscularly injected with an empty adenovirus vector; GD: mice intramuscularly injected with adenovirus vector Ad-TSHR289 and transplanted with fecal microbiota from GD patients; GD + BF: GD group mice orally supplemented with *B. fragilis*; GD + PA: GD group mice orally supplemented with propionate; GD + MMI + BF: GD + BF group mice treated with low-dose MMI; GD + MMI + PA: GD + PA group mice treated with low-dose MMI. (**H and I**) Comparison of intestinal abundance of *B. fragilis* (**H**) and propionate (**I**) among different groups of mice, *n* = 5. (**J**) Comparison of serum propionate levels among different groups of mice. *n* = 5. GD + MMI: GD group mice treated with low-dose MMI, other groups are the same as in E, F, and G. Statistical analysis was performed using one-way ANOVA with Tukey-HSD test. *P < 0.05; **P < 0.01; ***P < 0.001; ****P < 0.0001.

We observed a significant reduction in the abundance of *Bacteroides fragilis* in the gut microbiota of GD patients compared to healthy individuals. Notably, the reduced strains were predominantly *Bacteroides fragilis YCH46*, rather than the major pathogenic strain *Enterotoxigenic Bacteroides fragilis* (ETBF) (Fig. 2A and B). Furthermore, the levels of propionate in the serum and gut of GD patients were significantly decreased (Fig. 2C and D). MMI treatment failed to restore the reduced levels of *B. fragili*s and propionate in GD patients (Fig. 2A through D). Targeted metabolomics showed that propionate was one of the major metabolites of *B. fragilis* (Fig. S2A). Causal analysis indicated that changes in propionate levels are caused by *Bacteroides fragilis* abundance (Fig. S2B). The acidic environment of the stomach and the presence of various digestive enzymes in the small intestine could impair the viability of orally administered bacteria. Therefore, we examined the effects of oral supplementation with *B. fragilis* or propionate on the abundance of *B. fragilis* in the gut and the levels of propionate in the gut and serum. The results showed that oral supplementation with *B. fragilis*, either alone or in combination with MMI, significantly increased *B. fragilis* abundance in the gut of recipient mice and elevated propionate levels in both the gut and serum, indicating that orally administered *B. fragilis* effectively colonizes the gut and produces propionate. Propionate supplementation, whether alone or in combination with MMI, significantly increased propionate levels in the gut and serum but did not significantly affect the abundance of gut *B. fragilis* (Fig. S2C; Fig. 2E through G). MMI alone had no significant impact on the abundance of *B. fragilis* in the gut or propionate levels in the gut and serum of GD mice (Fig. 2H through J).

### Combination of *B. fragilis* or propionate with low-dose MMI improves thyroid function and reduces thyroid size in GD mice

**Fig 3:**
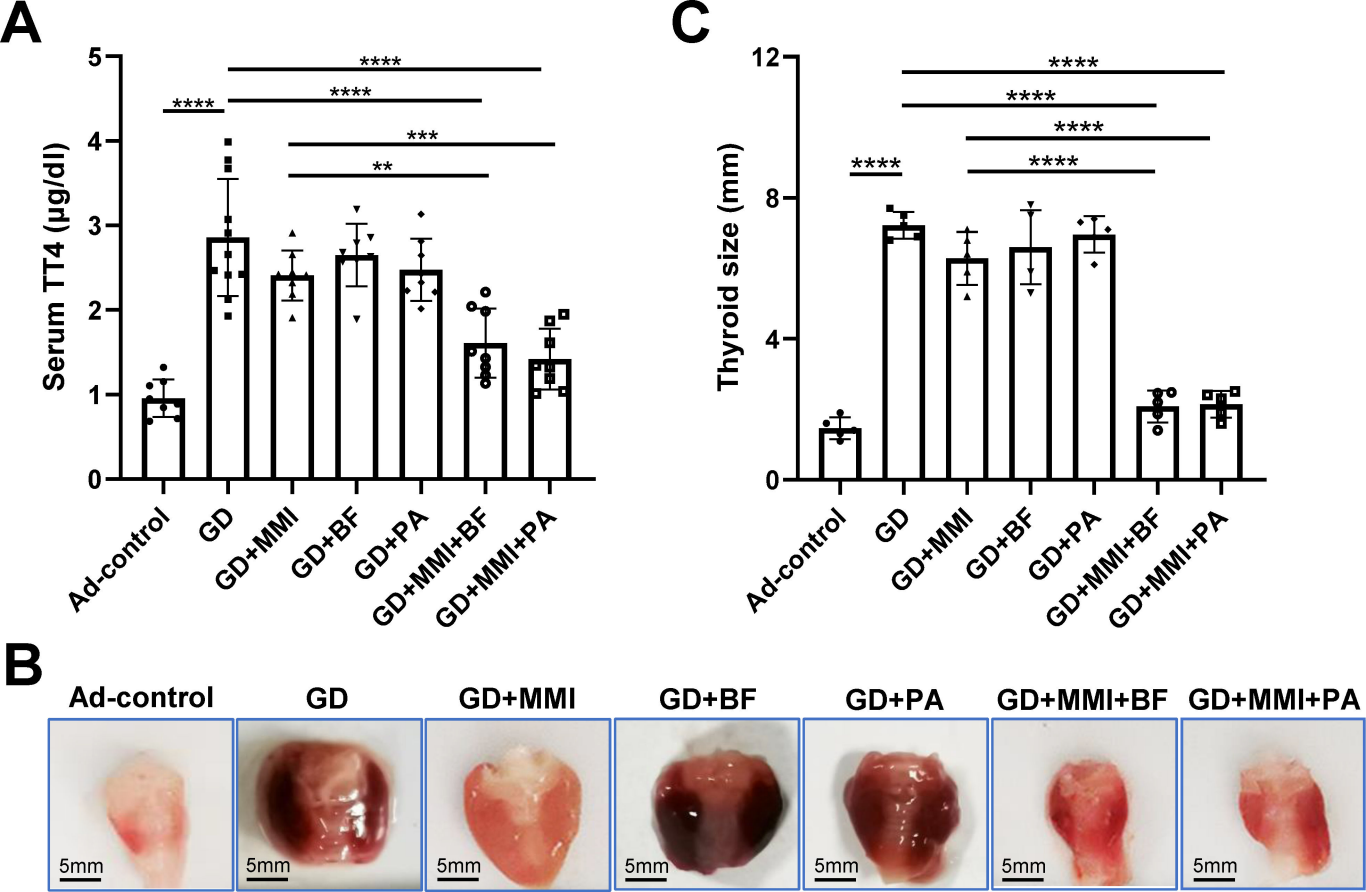
Serum TT4 levels and thyroid size in mice with different treatments. (**A**) Comparison of serum TT4 levels among different groups of mice. GD group: *n* = 11; other groups: *n* = 8. (**B**) Gross morphology of the thyroid gland in each group of mice. (**C**) Comparison of thyroid sizes among different groups of mice. *n* = 5. Ad-Control: mice intramuscularly injected with an empty adenovirus vector; GD: mice intramuscularly injected with adenovirus vector Ad-TSHR289 and transplanted with fecal microbiota from GD patients; GD + MMI: GD group mice treated with low-dose MMI; GD + BF: GD group mice orally supplemented with *B. fragilis*; GD + PA: GD group mice orally supplemented with propionate; GD + MMI + BF: GD + BF group mice treated with low-dose MMI; GD + MMI + PA: GD + PA group mice treated with low-dose MMI. Statistical analysis was performed using one-way ANOVA with the Tukey-HSD test. **P < 0.01; ***P < 0.001; ****P < 0.0001.

MMI is a first-line treatment for GD, but it can cause side effects such as allergic reactions, liver dysfunction, and hematopoietic damage (24–26). To mitigate these side effects, we investigated whether combining *B. fragilis* or propionate with MMI could enhance the therapeutic efficacy of MMI on thyroid function and morphology while reducing the required dosage of MMI in GD mice. Our results showed that GD mice exhibited significantly elevated serum TT4 levels compared to control mice (Ad-control). While low-dose MMI, *B. fragilis*, or propionate alone reduced serum TT4 levels in GD mice, these reductions were not statistically significant. However, the combination of low-dose MMI with either *B. fragilis* or propionate significantly reduced serum TT4 levels, thereby improving hyperthyroidism in GD mice (Fig. 3A). In addition, we observed a significant enlargement of the thyroid in GD mice. Neither low-dose MMI, *B. fragilis*, nor propionate alone had a significant effect on thyroid size. However, the combination of low-dose MMI with either *B. fragilis* or propionate significantly reduced thyroid size in GD mice (Fig. 3B and C). Moreover, heat inactivation significantly attenuated the effects of *B. fragilis*, either alone or in combination with MMI, on improving thyroid function in GD mice (Fig. S2D). These results suggest that the combination of low-dose MMI with live *B. fragilis* or propionate has a significant synergistic effect in improving hyperthyroidism and reducing thyroid size in GD.

### Combination of *B. fragilis* or propionate with low-dose MMI reduces systemic inflammation and autoimmunity against TSHR in GD mice

**Fig 4:**
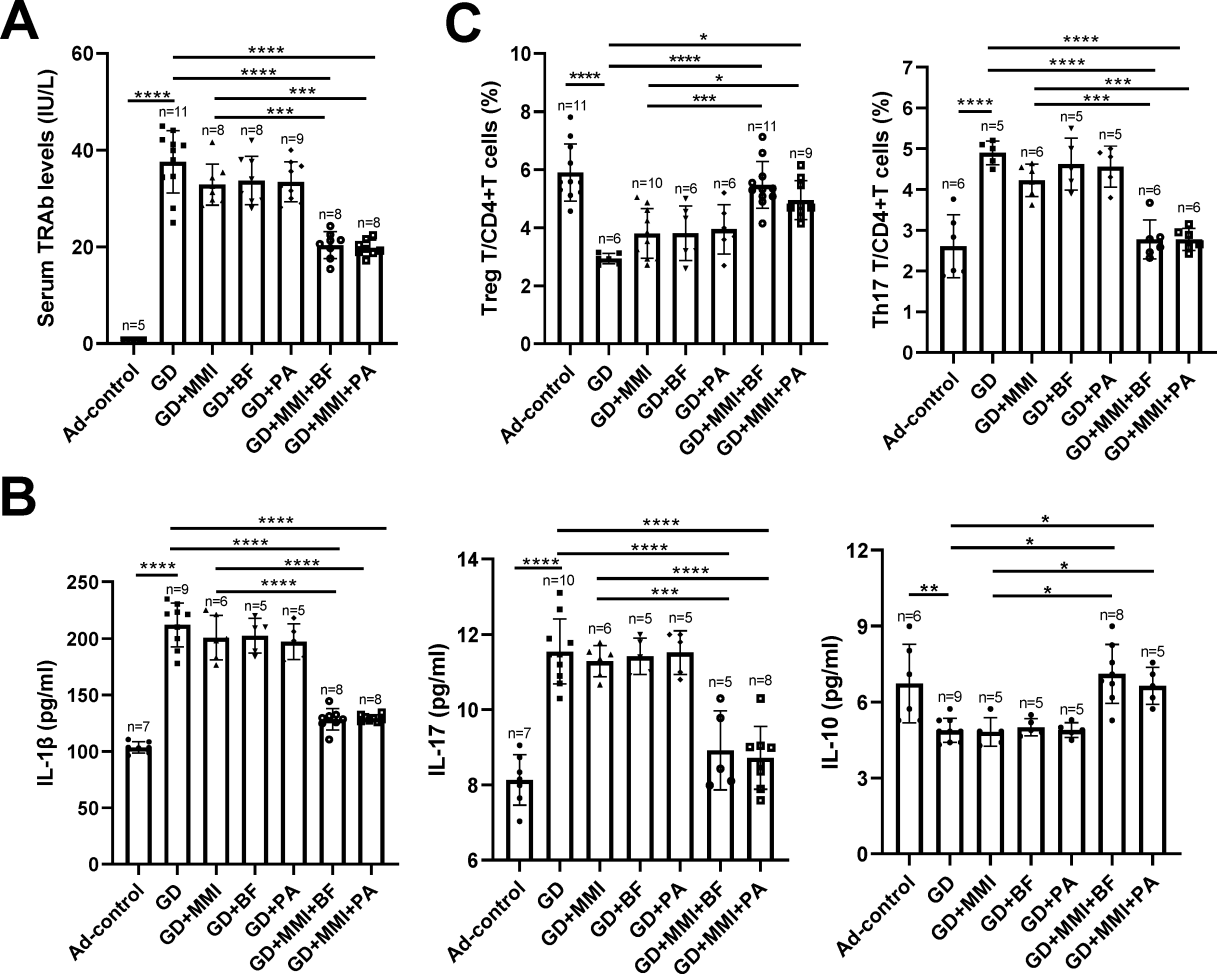
Therapeutic Effects of *B. fragilis*, propionate, and low-dose MMI alone or in combination with GD mice. (**A and B**) Comparison of serum TRAb (**A**) and cytokine levels (**B**) among different groups of mice. (**C**) Comparison of Treg and Th17 cell proportions in peripheral blood CD4+ T cells among different groups of mice. The sample size for each group is indicated in the figure. Ad-Control: mice intramuscularly injected with an empty adenovirus vector; GD: mice intramuscularly injected with adenovirus vector Ad-TSHR289 and transplanted with fecal microbiota from GD patients; GD + MMI: GD group mice treated with low-dose MMI; GD + BF: GD group mice orally supplemented with *B. fragilis*; GD + PA: GD group mice orally supplemented with propionate; GD + MMI + BF: GD + BF group mice treated with low-dose MMI; GD + MMI + PA: GD + PA group mice treated with low-dose MMI. Statistical analysis was performed using one-way ANOVA followed by the Tukey-HSD test. *P < 0.05; **P < 0.01; ***P < 0.001; ****P < 0.0001.

Systemic inflammation and TSHR autoimmunity are key pathological features of GD. Therefore, we investigated the effects of low-dose MMI, *B. fragilis*, and propionate, either alone or in combination, on systemic inflammation and TSHR autoimmunity in GD mice. Compared to control mice (Ad-control), GD mice exhibited significantly elevated serum levels of TRAb and inflammatory cytokines (IL-1β and IL-17), while levels of anti-inflammatory IL-10 were significantly reduced (Fig. 4A and B). In addition, GD mice had a significantly decreased proportion of CD4 + CD25 + FOXP3+ T cells (Tregs) and an increased proportion of CD4+IL17+ Th17 cells in peripheral CD4+ T cells (Fig. 4C). Low-dose MMI, *B. fragilis*, or propionate alone did not significantly affect serum levels of TRAb or pro-inflammatory and anti-inflammatory cytokines in GD mice. However, the combination of low-dose MMI with either *B. fragilis* or propionate significantly reduced serum levels of TRAb and pro-inflammatory cytokines (IL-1β and IL-17) while increasing the level of the anti-inflammatory cytokine IL-10. In addition, these combinations significantly reduced the Th17/CD4+ T-cell ratio and increased the Treg/CD4+ T-cell ratio in the peripheral blood of GD mice (Fig. 4A through C). However, the above synergistic effects of inactivated *B. fragilis* and MMI in reducing autoimmunity against TSHR in GD mice were abolished (Fig. S2E). These findings indicate that the combination of low-dose MMI with live *B. fragilis* or propionate synergistically improves systemic inflammation and TSHR autoimmunity in GD mice.

### Combination of *B. fragilis* or propionate with low-dose MMI reduces thyroid inflammation in GD mice

**Fig 5:**
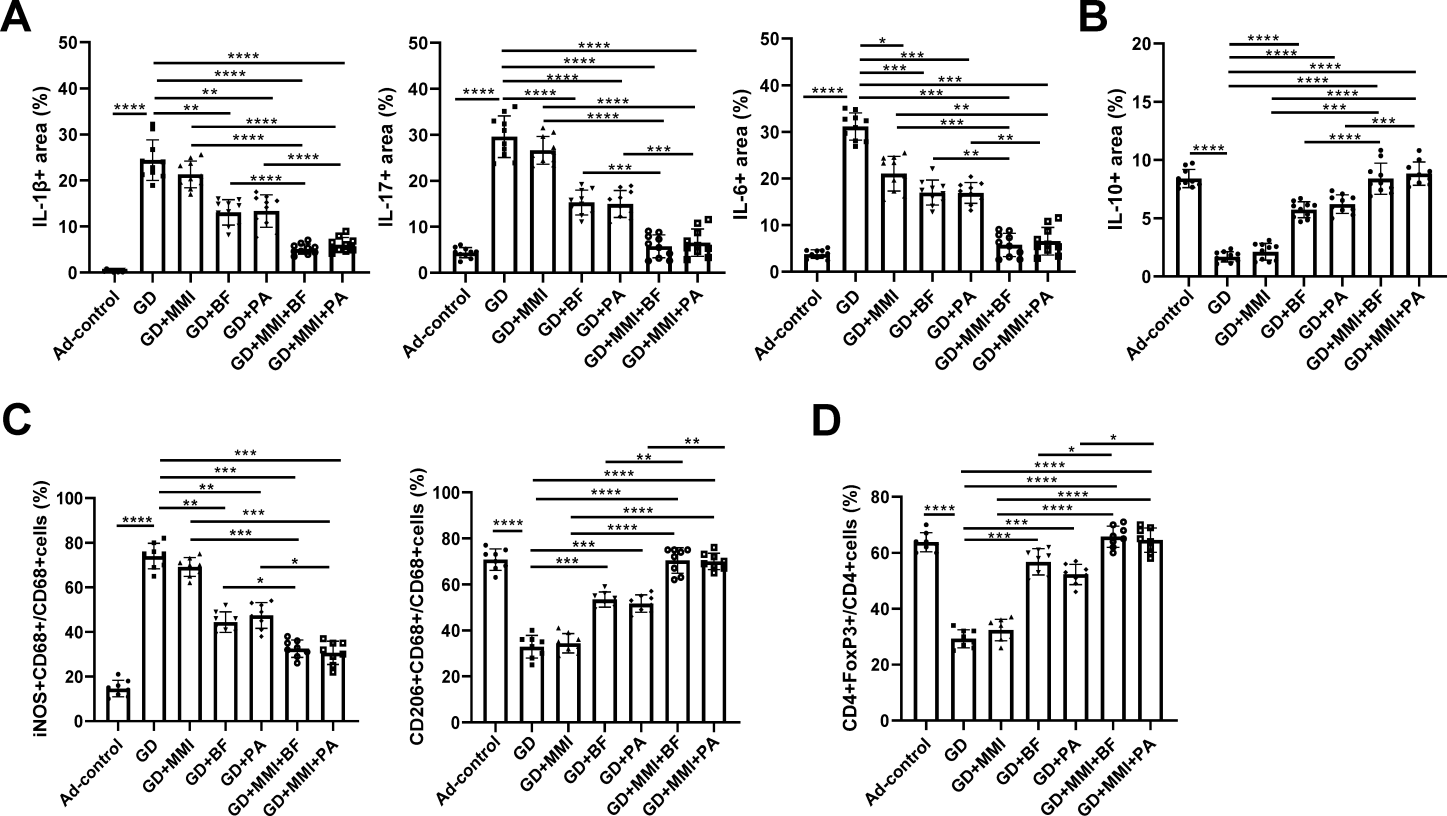
Effects of *B. fragilis*, propionate, and low-dose MMI alone or in combination on cytokine levels, Treg cells, and macrophage subpopulations in thyroid tissues of GD mice. (**A and B**) Comparison of the levels of pro-inflammatory cytokines (**A**) and anti-inflammatory cytokine IL-10 (**B**) in thyroid tissues among different groups of mice. *n* = 10. (**C and D**) Comparison of the proportions of M1 (iNOS+CD68+) and M2 (CD206+CD68+) macrophages (**C**) and Treg cells (CD4+FoxP3+) (**D**) in thyroid tissues among different groups of mice. *n* = 8. Ad-Control: mice intramuscularly injected with an empty adenovirus vector; GD: mice intramuscularly injected with adenovirus vector Ad-TSHR289 and transplanted with fecal microbiota from GD patients; GD + MMI: GD group mice treated with low-dose MMI; GD + BF: GD group mice orally supplemented with *B. fragilis*; GD + PA: GD group mice orally supplemented with propionic acid; GD + MMI + BF: GD + BF group mice treated with low-dose MMI; GD + MMI + PA: GD + PA group mice treated with low-dose MMI. Statistical analysis was performed using one-way ANOVA with the Tukey-HSD test. *P < 0.05; **P < 0.01; ***P < 0.001; ****P < 0.0001.

The enhanced inflammatory response and inflammatory cell infiltration in thyroid tissue are key pathological features of GD. We investigated the effects of low-dose MMI, *B. fragilis*, and propionate, either alone or in combination, on thyroid inflammation in GD mice. The results showed that, compared to euthyroid mice (Ad-control), GD mice exhibited significantly elevated levels of pro-inflammatory cytokines (IL-1β, IL-17, and IL-6) and reduced levels of the anti-inflammatory cytokine (IL-10) in thyroid tissue, indicating an enhanced inflammatory response (Fig. 5A and B; Fig. S3). Supplementation with *B. fragilis* or propionate alone significantly reduced the levels of IL-1β, IL-17, and IL-6 and increased IL-10 levels in thyroid tissue. Low-dose MMI alone significantly decreased IL-6 levels in the thyroid tissue of GD mice but had no significant effect on other cytokine levels (Fig. 5A and B; Fig. S3). However, the combination of low-dose MMI with either *B. fragilis* or propionate significantly reduced the levels of IL-1β, IL-6, and IL-17, and increased IL-10 levels with greater efficacy than either treatment alone (Fig. 5A and B; Fig. S3).

In addition, we found that compared to euthyroid mice (Ad-control), GD mice exhibited a significant increase in the proportion of pro-inflammatory M1 macrophages (iNOS+CD68+) in thyroid tissue, along with a significant decrease in the proportions of anti-inflammatory M2 macrophages (CD206+CD68+) and Treg cells (FOXP3+CD4+). Supplementation with *B. fragilis* or propionate significantly alleviated the aforementioned abnormalities in GD mice, while low-dose MMI alone did not significantly affect these cell populations. However, the combination of low-dose MMI with either *B. fragilis* or propionate significantly reduced the proportion of M1 macrophages and increased the proportions of M2 macrophages and Treg cells in thyroid tissue of GD mice, with better efficacy than either treatment alone (Fig. 5C and D; Fig. S4). These results suggest that the combination of low-dose MMI with *B. fragilis* or propionate markedly attenuates inflammation in the thyroid tissue of GD mice and improves their immune status.

## DISCUSSION

This study demonstrates that oral supplementation with *B. fragilis* or propionate can effectively enhance the efficacy of low-dose MMI in ameliorating hyperthyroidism, inflammation, and autoimmunity in GD mice. The combination treatment significantly improves thyroid function, reduces thyroid size, and mitigates both systemic and local inflammation. These findings highlight the potential of microbiota-based adjuvant therapies to enhance current GD treatments, offering a promising avenue for reducing drug dosages and minimizing side effects.

Over the past decade, microbiota-targeted therapies, including probiotic/prebiotic supplementation and FMT, have been successfully applied with considerable success to treat various diseases, such as infectious diseases (e.g., *Clostridium difficile* infection), gastrointestinal disorders (e.g., ulcerative colitis and Crohn’s disease), and metabolic diseases (e.g., diabetes and obesity) (27, 28). Given that gut dysbiosis is a pivotal etiological factor in autoimmune diseases, interventions aimed at correcting this imbalance represent a novel therapeutic avenue for treating autoimmune diseases, including GD. For example, studies have demonstrated that the probiotic VSL#3 effectively alleviates symptoms in patients with ulcerative colitis. FMT promotes mucosal healing and ameliorates clinical manifestations of Crohn’s disease. Probiotics such as *Lactobacillus acidophilus* and *Bifidobacterium bifidum* have been shown to modulate immune activity, decrease the production of inflammatory cytokines, and alleviate inflammatory responses in rheumatoid arthritis. Moreover, *Lactobacillus rhamnosus* has been found to significantly reduce asthma symptoms and enhance lung function. Probiotic interventions can also reduce disease activity in systemic lupus erythematosus, improve patients’ quality of life, and diminish autoantibody production (21–23). Compared to traditional immunosuppressive therapies for autoimmune diseases, microbiota-targeted therapies offer unique advantages. Conventional therapies generally aim to control disease progression by broadly suppressing immune responses, which may lead to an increase in the risks of infections and malignancies (29). By contrast, microbiota-targeted therapies leverage the natural regulatory effects of commensal microbes, which are more harmonious with the body’s self-regulation and minimize excessive immunosuppression, thus offering better safety and tolerability for long-term use. By restoring immune homeostasis through the modulation of gut microbiota balance, microbiota-targeted therapies have the potential for sustained remission and reduced relapse rates. Moreover, because the composition of the gut microbiota varies significantly among individuals, microbiota-targeted therapies can be personalized based on the characteristics of an individual’s gut microbiota, further enhancing therapeutic efficacy and minimizing side effects.

Current standard treatments for GD include antithyroid drugs, radioactive iodine ablation, and surgical resection (4). Despite their effectiveness, these therapies have significant drawbacks, such as adverse effects, high recurrence rates, and complications including permanent hypothyroidism (5–8). Our previous studies have demonstrated that gut microbiota dysbiosis is a critical cause and pathological feature of GD (19). To better simulate the pathological characteristics of GD patients, we transplanted gut microbiota from GD patients to the classical GD mouse model. This modified model recapitulated the key inflammatory features of GD patients, including significantly altered levels of pro- and anti-inflammatory cytokines (e.g., IL-1β, IL-6, IL-17, and IL-10). The results demonstrated the reliability and relevance of this mouse model for studying the pathogenesis of GD and evaluating therapeutic interventions. It also provides a foundation for assessing the therapeutic efficacy of *Bacteroides fragilis* and its metabolite, propionate. Based on the mice model, our study demonstrates that *B. fragilis* and its metabolite, propionate, effectively ameliorate hyperthyroidism in GD mice, reduce inflammatory responses and TSHR autoimmunity, and promote immune homeostasis. Moreover, when combined with MMI, *B. fragilis* or propionate not only did it significantly enhance the suppression of both local and systemic thyroid inflammation, but it also effectively reduced the required dose of MMI, thereby mitigating its side effects and facilitating long-term use. This synergistic effect offers a potential strategy for optimizing GD treatment. We found that low-dose MMI reduced IL-6 levels in thyroid tissues of GD mice, exerting a localized anti-inflammatory effect, but it did not impact systemic inflammation. This is because MMI primarily acts by inhibiting thyroid peroxidase (TPO), thereby blocking excessive thyroid hormone synthesis and alleviating thyroid tissue inflammation (7). In addition, we observed that low-dose MMI had minimal therapeutic effects in GD mice. This can be attributed to the MMI dose used in this study, which was approximately 1/18 of the clinically equivalent therapeutic dose (30). The lack of significant efficacy with low-dose MMI should be therefore reasonable (31, 32). Nevertheless, the combination of low-dose MMI with *Bacteroides fragilis* or propionate (PA) achieved significantly enhanced therapeutic outcomes compared to monotherapy. This effect is likely due to their synergistic immunomodulatory mechanisms. *Bacteroides fragilis* and PA regulate systemic immunity by promoting the differentiation of regulatory T cells (Tregs), modulating pro-inflammatory cytokine levels (e.g., IL-1β and IL-6), and restoring immune homeostasis, thereby compensating for the limited localized anti-inflammatory effects of MMI in thyroid tissue. Furthermore, *B. fragilis* and propionate are naturally occurring bacteria and metabolites in healthy intestines, exhibiting fewer side effects compared to traditional therapies. Therefore, *B. fragilis* and propionate warrant further investigation for the treatment of GD.

As a next-generation probiotic, *B. fragilis* can promote the differentiation and function of Tregs and macrophages, reduce inflammatory reactions, and restore the Treg/Th17 balance through its metabolic products such as polysaccharide A and SCFAs, thereby maintaining immune tolerance (33–35). In patients with autoimmune diseases such as inflammatory bowel disease and rheumatoid arthritis, the abundance of gut *B. fragilis* is significantly reduced and closely related to disease severity (36). Supplementation with *B. fragilis* effectively alleviates intestinal inflammation in mouse models of colitis (37). However, in some autoimmune disease patients, increased levels of *B. fragilis* have been reported to exacerbate inflammation, which might be due to disease-specific factors, individual microbiota variability, or strain-specific differences in *B. fragilis* (38). Propionate is an important metabolite of *B. fragilis* and has anti-inflammatory and immunomodulatory effects. It has been demonstrated to increase the number of Tregs in the colon and upregulate Foxp3 and IL-10 expression (39, 40). Propionate also enhances intestinal mucosal barrier function by increasing the expression and secretion of MUC2 in goblet cells. In monocytes, propionate can effectively inhibit LPS-induced TNFα and nitric oxide (NO) production, thereby reducing NF-κB signaling activity. However, some studies have reported that the infusion of sodium propionate into the pig cecum significantly increases the abundance of *Bacteroidetes* and upregulates the expression of NF-κB and IL-18, thereby enhancing inflammatory responses (41). Our study demonstrates that *B. fragilis* and its metabolite, propionate, either alone or in combination with low-dose MMI, can significantly ameliorate immune dysregulation, reduce inflammatory responses, and improve thyroid function in GD mice. The underlying mechanism may involve the promotion of Treg cell differentiation and function through PSA and propionate, thereby modulating inflammation and autoimmunity in thyroid tissue. However, *B. fragilis* also produces butyric acid, and whether this metabolite contributes to the effects of *B. fragilis* on GD requires further investigation.

In conclusion, our study demonstrates that oral supplementation with *B. fragilis* or propionate can significantly ameliorate the condition of GD mice. When combined with low-dose MMI, they exert synergistic anti-inflammatory and immunomodulatory effects, thereby improving thyroid function. This combination allows for a reduction in MMI dosage without compromising therapeutic efficacy. These findings provide novel insights and targets for GD treatment from a gut microbiome perspective, potentially reducing adverse reactions and enhancing the management of GD.

## MATERIALS AND METHODS

### Subject recruitment and fecal sample collection

A total of 30 newly diagnosed and untreated patients with GD were recruited from Shandong Provincial Hospital, following diagnostic criteria based on the 2018 European Thyroid Association Guideline for the Management of Graves’ Hyperthyroidism. Exclusion criteria included pregnancy, smoking, alcohol dependence, diarrhea, hypertension, diabetes, hyperlipidemia, and recent (within 3 months) use of medications such as antibiotics, probiotics, prebiotics, synbiotics, hormonal drugs, laxatives, proton pump inhibitors, amiodarone, or herbal remedies. Patients with a history of autoimmune diseases, malignant tumors, gastrointestinal surgery, radioisotope therapy for GD, or moderate to severe ophthalmopathy were also excluded. Collected fecal samples were immediately frozen in liquid nitrogen and subsequently stored at −80°C.

### Construction and activity verification of adenoviral vectors

Recombinant adenovirus vector Ad-TSHR289 and the corresponding empty vector were obtained from Hanheng Biotechnology Co., Ltd. To verify the activity of these vectors, 293T cells were infected with them. After 48 hours of incubation, total protein was extracted, and Western blot analysis was performed to detect the expression of green fluorescent protein (GFP) and thyroid-stimulating hormone receptor (TSHR).

### FMT and GD mouse model construction

Sixty-five female BALB/c mice (6- to 8-week-old) were purchased from Beijing Sipeifu Biotechnology Co., Ltd. Mice were housed under standard conditions, with a 12 hour light/dark cycle and *ad libitum* access to food and water. For gut microbiota depletion, mice were gavaged daily for 5 days with 10 mg each of antibiotics (ampicillin, neomycin sulfate, metronidazole, and vancomycin). Subsequently, antibiotics were added to the drinking water at concentrations of 1 g/L for ampicillin, neomycin sulfate, and metronidazole, and 500 mg/L for vancomycin for an additional 2 weeks. After antibiotic treatment, mice were gavaged daily with 200 µL of fecal microbiota suspension from a sample pool of 30 GD patients for 1 week, and then every other day for 9 weeks. Subsequently, mice were intramuscularly injected with Ad-TSHR1-289 or empty vectors every 3 weeks for a total of three injections. After successful model establishment, GD mice were gavaged once every 3 days with methimazole (1.2 mg/kg/day) (30), *Bacteroides fragilis YCH46*, or sodium propionate either alone or in combination. In addition, GD mice transplanted with fecal microbiota from GD patients served as the control group for evaluating therapeutic efficacy, while the Ad-Control group of mice intramuscularly injected with the empty vector was used as the negative control, and they were gavaged with PBS. After 12 weeks, serum, thyroid tissue, and fecal samples were collected from each group. Serum levels of IL-1β, IL-6, IL-17, IL-10, TT4, and TRAb were measured using ELISA.

### Immunohistochemistry and immunofluorescence staining

Thyroid tissues were collected from each group of mice, photographed, and fixed in 4% paraformaldehyde. Fixed tissues were dehydrated, embedded in paraffin, and sectioned at a thickness of 5 µm. Sections were deparaffinized, dehydrated, and subjected to antigen retrieval. After treatment with 3% hydrogen peroxide for 10 min and PBS washing, sections were incubated with goat serum at room temperature for 1 hour. Some sections were incubated overnight at 4°C with primary antibodies against IL-1β (1:300), IL-6 (1:100), IL-17 (1:300), or IL-10 (1:50). After PBS washing, sections were incubated with secondary antibodies at room temperature for 1 hour, followed by DAB staining. For immunofluorescence, sections were incubated overnight at 4°C with primary antibodies against iNOS, CD68+, CD206, FoxP3, and CD4. Following PBS washing under light-protected conditions, the corresponding secondary antibodies were applied for 2 hours at room temperature. After additional PBS washing, DAPI was used for nuclear staining. Sections were then imaged using optical and fluorescence microscopes. For IF, the regions of interest (ROI) in fluorescence microscopy images of thyroid tissues were selected, and the mean gray value and signal intensity were measured. For IHC, staining signals were separated using Color Deconvolution, and the positive area and staining intensity were quantified. Quantitative analysis was performed using ImageJ software. Antibodies used in this study were listed in Table S1.

### Western blot analysis

Cells were harvested, washed with pre-cooled PBS, and lysed in RIPA buffer for 30 minutes. Lysates were centrifuged at 10,000 rpm for 15 minutes at 4°C, and the supernatant was collected as purified total protein. Protein concentration was measured using the BCA assay. Subsequently, 50 µg protein was mixed with sample loading buffer and heated at 99°C for 5 minutes. Proteins were separated by SDS-PAGE and electrotransferred onto PVDF membranes. Membranes were blocked with non-fat dry milk (50 mg per mL of PBS) at room temperature for 2 hours, washed with TBST, and incubated with primary antibodies overnight at 4°C. After TBST washing, membranes were incubated with secondary antibodies at room temperature for 90 minutes. Protein bands were visualized using an enhanced chemiluminescent detection kit. Images were captured using the ChemiDoc XRS + imaging system, and quantitative analysis was performed with Image J software. Antibodies used are listed in Table S1.

### ELISA detection of serum inflammatory cytokines

Peripheral blood from mice was stood at 4°C for 4 hours, followed by centrifugation at 3,500 rpm for 10 minutes at 4°C. The resulting serum was collected, and the levels of TT4, TRAb, IL-1β, IL-6, IL-17, and IL-10 were measured using commercially available ELISA kits following the manufacturer’s instructions.

### Fecal genomic DNA extraction and real-time quantitative PCR (qPCR)

Genomic DNA was extracted from 200 mg fecal samples using 2 × CTAB buffer and phenol-chloroform as previously described (27). DNA concentration and purity were assessed using a NanoDrop 2000 (Thermo Scientific). PCR amplification was performed using TB Green Premix Ex Taq, specific primers, and a LightCycler 480 system (Roche, Basel, Switzerland). Relative gene expression was calculated using the 2−ΔΔCt method. Primers used in this study were listed in Table S2.

### Flow cytometry assay

Mouse peripheral blood mononuclear cells (PBMCs) were purified using Ficoll gradient centrifugation. Cells were incubated with PMA, ionomycin, and a protein transport inhibitor for 5 hours at 37°C in a 5% CO2 atmosphere. After incubation, cells were collected by centrifugation and adjusted to a concentration of 10^7 cells/mL. Subsequently, cells were stained in the dark for 1 hour with CD4-FITC, FoxP3-PE, CD25-APC, or IL-17-PE antibodies in a permeabilization buffer. Cells were then centrifuged at 300 g for 5 min and washed twice with PBS. Finally, cells were resuspended in a flow cytometry buffer and analyzed using an Agilent NovoCyte flow cytometer.

### Assay of serum propionate levels

Serum samples (50 µL) were mixed with an equal volume of methanol and vortexed thoroughly. Fecal samples (100 mg) were homogenized in 1 mL of pre-cooled methanol and sonicated for 10 minutes. Samples were centrifuged at 12,000 rpm for 10 minutes at 4°C, and the supernatants were collected for analysis. Each supernatant (50 µL) was mixed with an internal standard and N-tert-butyldimethylsilyl trifluoroacetamide (BSTFA, 50 µL) containing 1% TMCS, followed by incubation at 60°C for 1 hour. The derivatized samples (1 µL) were analyzed using a Shimadzu GC-2030 gas chromatograph coupled to a Shimadzu TQ8050 NX mass spectrometer. Chromatographic separation was performed on an HP-5MS column (30 m × 0.25 mm × 0.25 µm). Data analysis was conducted using Shimadzu GCMSsolution software. Propionate levels were quantified using the internal standard method.

### Cultivation of *Bacteroides fragilis YCH46*

*B. fragilis YCH46* was obtained from Beijing Zhongke Quality Inspection Biotechnology Co., Ltd. Modified Gifu Anaerobic Medium (GAM) broth was prepared according to the manufacturer’s instructions, sterilized at 121°C for 15 minutes, and pre-reduced in an anaerobic chamber for 24 hours. The frozen *B. fragilis YCH46* was thawed and inoculated onto a pre-reduced GAM solid medium, followed by anaerobic incubation at 37°C for 48 hours to obtain single colonies. A single colony was picked and inoculated into 10 mL of pre-reduced GAM liquid medium, then incubated anaerobically at 37°C overnight to reach the logarithmic growth phase. Subsequently, 1 mL of the bacterial culture was transferred into 100 mL of pre-reduced GAM liquid medium and incubated anaerobically at 37°C until the OD_600_ reached 0.6–0.8. This culture was used for subsequent experiments.

### Statistical analysis

The Shapiro-Wilk test was used to assess the normality of data distribution. Data following normal distribution were presented as mean ± standard deviation (x̄ ±s). Comparisons between two groups were performed using a two-tailed t-test, while comparisons among multiple groups were conducted using one-way ANOVA. For non-normally distributed data, the Wilcoxon rank-sum test was used for comparisons between two groups, and the Kruskal-Wallis test was applied for multiple group comparisons. Statistical significance was defined as *P*-value or adjusted *P*-value < 0.05. All statistical analyses were conducted using GraphPad Prism 6.0 and R software. All experiments were performed at least in triplicate.

## Data Availability

All data supporting the findings of this study are available within the article and its supplementary materials. Data from 16S rRNA gene sequencing have been deposited in the Genome Sequence Archive (GSA) at the China National GeneBank DataBase (CNGBdb) under GSA code CRA023689.

## SUPPLEMENTAL MATERIAL

Fig. S1 spectrum.03186-24-s0001.tif GD Mouse model construction flowchart and verification of adenovirus vector activity and FMT.

Fig. S2 spectrum.03186-24-s0002.tif Metabolite detection in *B. fragilis* cultures, causal analysis, and animal intervention experiment flowchart and efficacy comparison of inactive and live *B. fragilis*.

Fig. S3 spectrum.03186-24-s0003.tif Immunohistochemistry staining of inflammatory cytokines in thyroid tissues of mice.

Fig. S4 spectrum.03186-24-s0004.tif Immunofluorescence staining of M1 and M2 macrophages and Treg cells in thyroid tissues of mice.

Supplemental material spectrum.03186-24-s0005.docx Supplemental figure legends; Tables S1 and S2.

ASM does not own the copyrights to Supplemental Material that may be linked to, or accessed through, an article. The authors have granted ASM a non-exclusive, world-wide license to publish the Supplemental Material files. Please contact the corresponding author directly for reuse.
