# Supplementary figures and images for: Bacteroides fragilis and propionate synergize with low-dose methimazole to treat Graves’ disease
Source: Microbiol Spectr. 2025 Apr 23;13(6):e03186-24. doi: 10.1128/spectrum.03186-24 (PMC12131849; doi:10.1128/spectrum.03186-24)

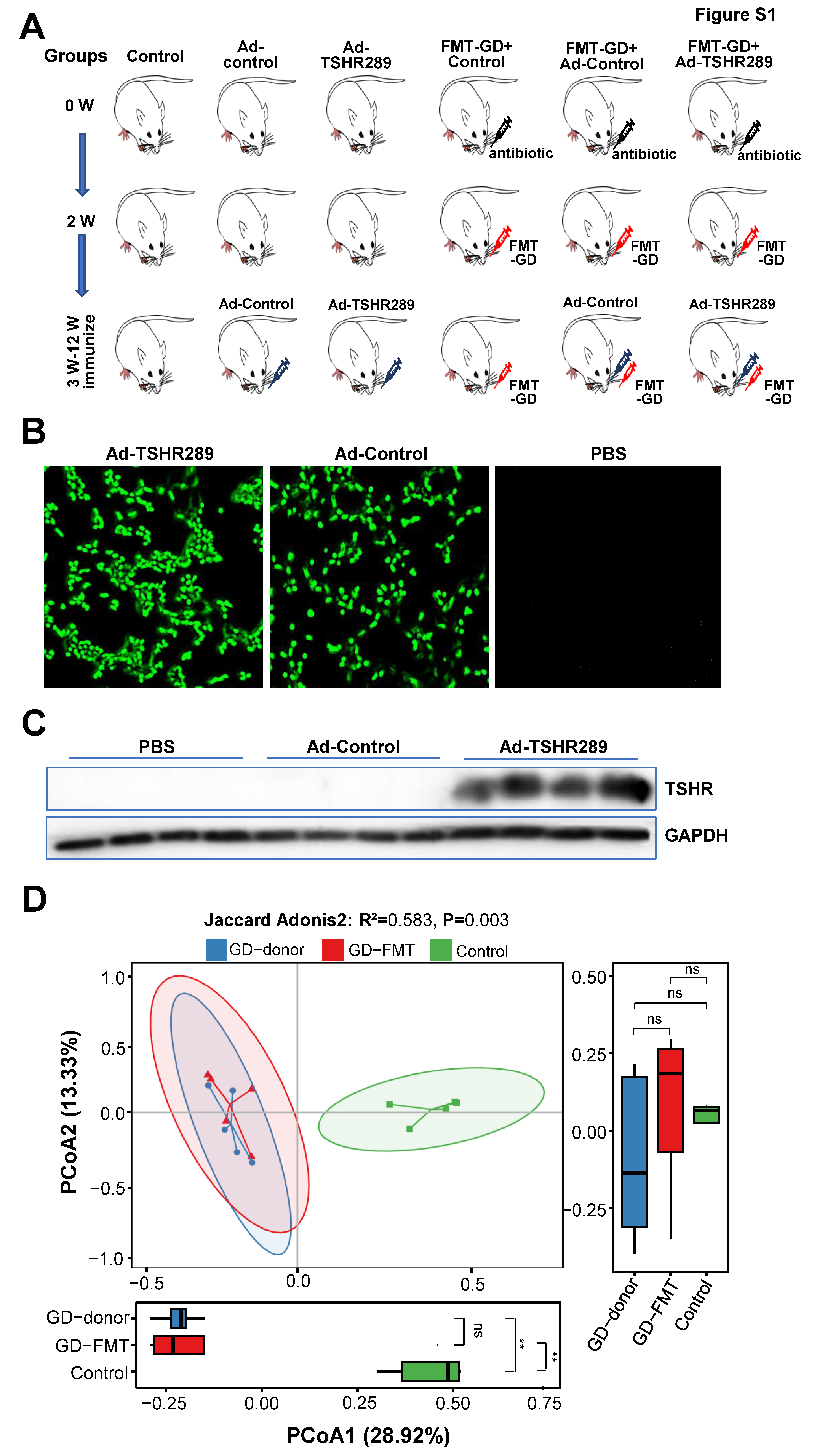

Supplement: Fig. S1 — GD Mouse model construction flowchart and verification of adenovirus vector activity and FMT. [file spectrum.03186-24-s0001.tif]

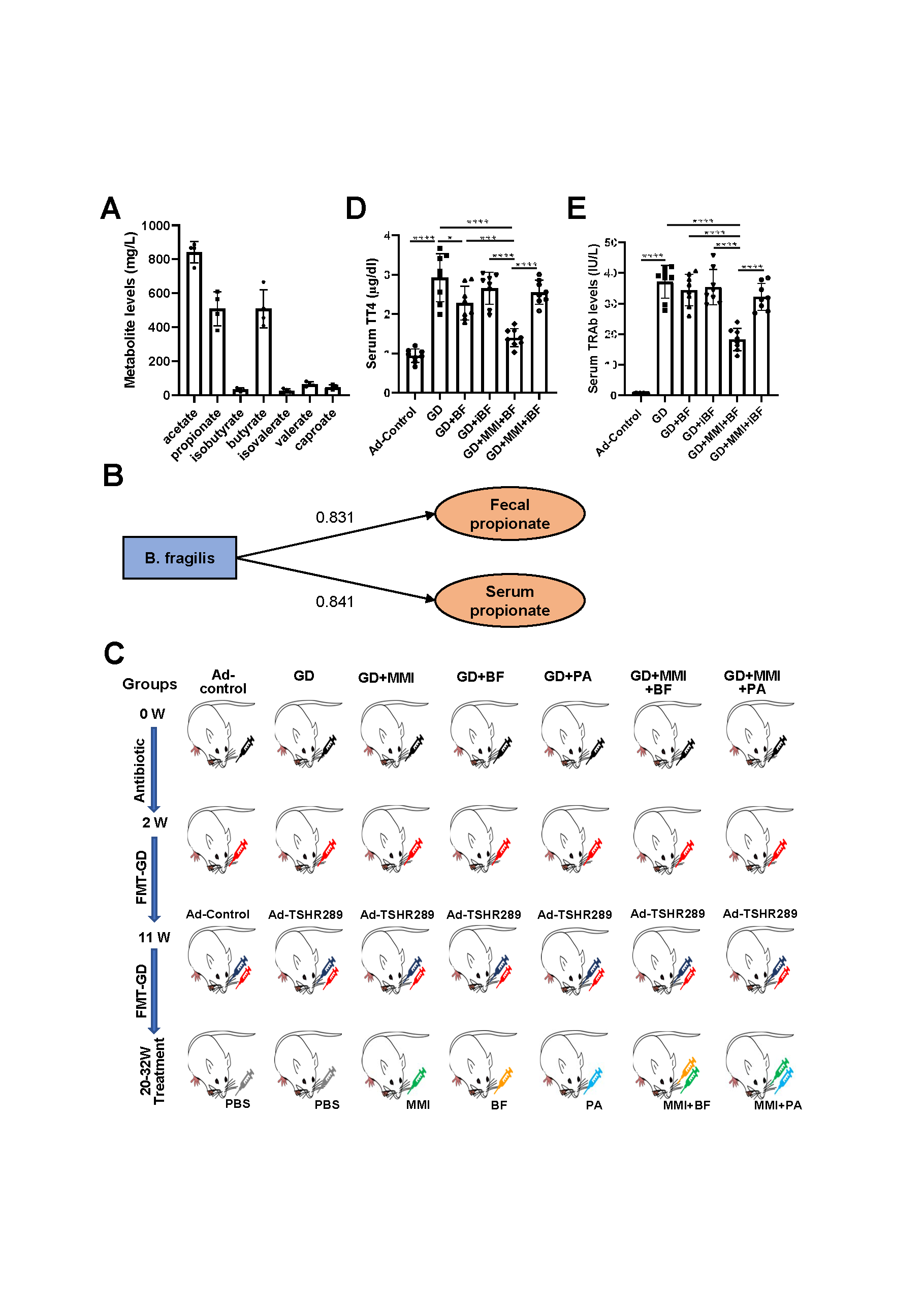

Supplement: Fig. S2 — Metabolite detection in B. fragilis cultures, causal analysis, and animal intervention experiment flowchart and efficacy comparison of inactive and live B. fragilis. [file spectrum.03186-24-s0002.tif]

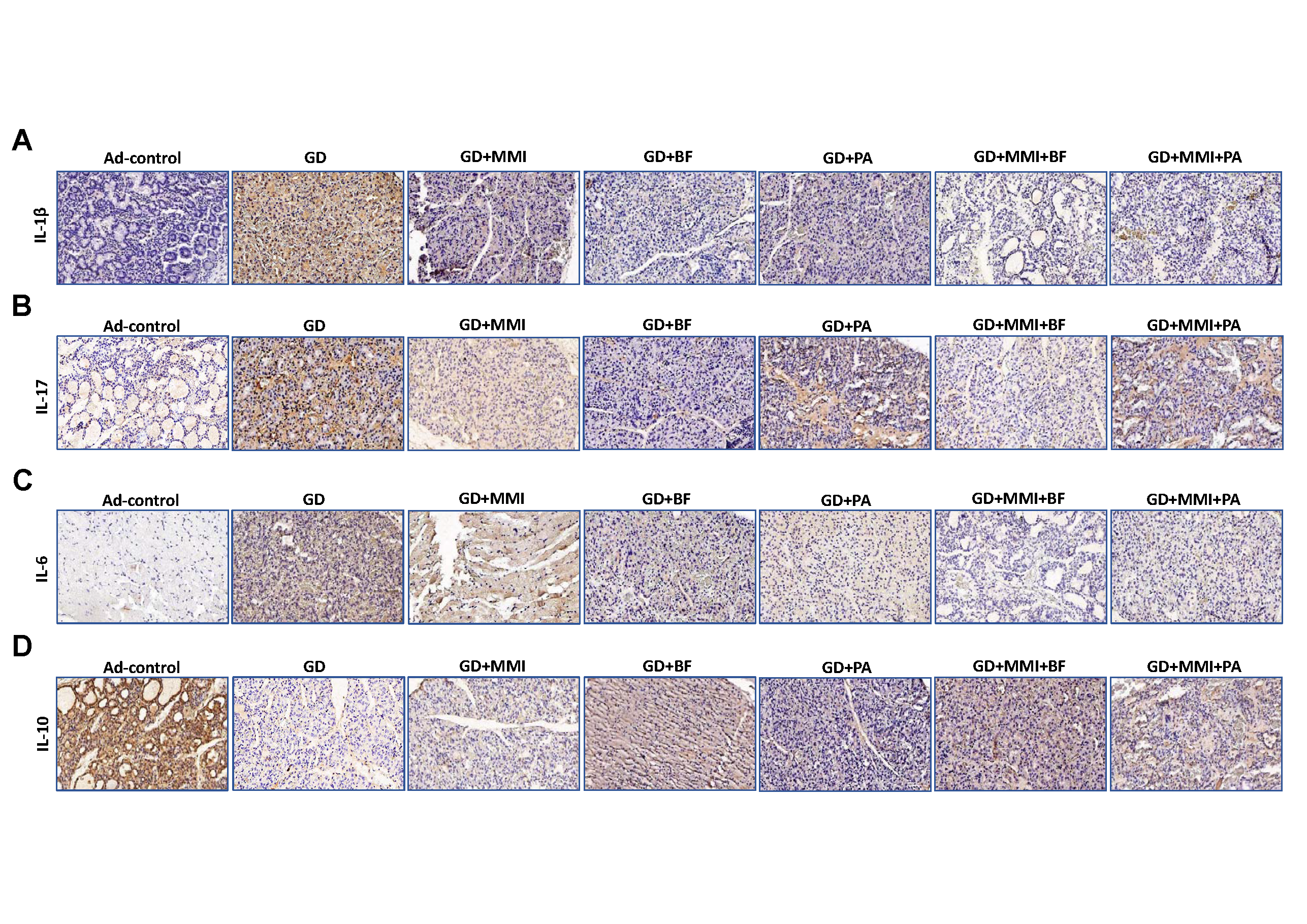

Supplement: Fig. S3 — Immunohistochemistry staining of inflammatory cytokines in thyroid tissues of mice. [file spectrum.03186-24-s0003.tif]

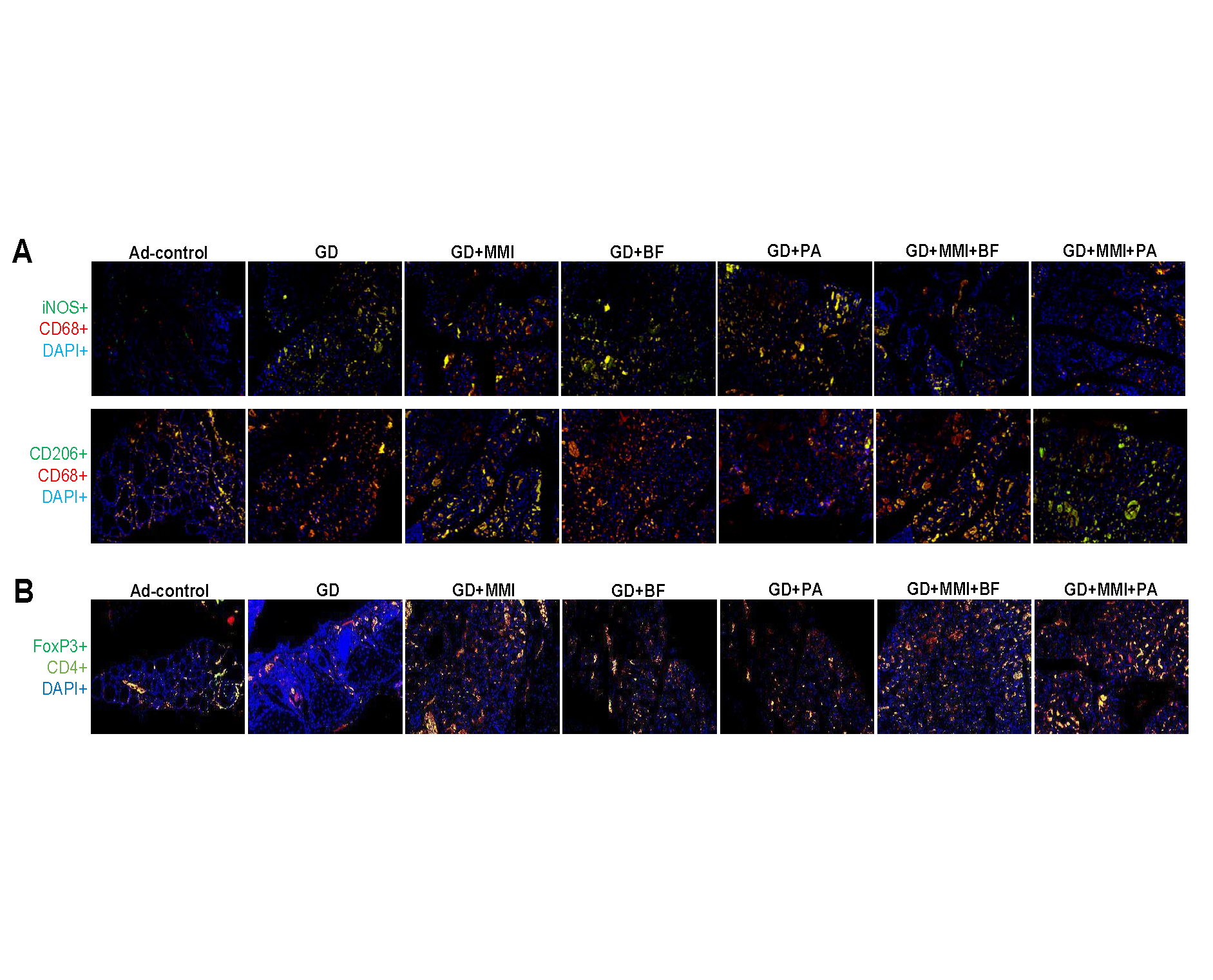

Supplement: Fig. S4 — Immunofluorescence staining of M1 and M2 macrophages and Treg cells in thyroid tissues of mice. [file spectrum.03186-24-s0004.tif]
