# Supplementary material for: Bacteroides fragilis and propionate synergize with low-dose methimazole to treat Graves’ disease
Source: Microbiol Spectr. 2025 Apr 23;13(6):e03186-24. doi: 10.1128/spectrum.03186-24 (PMC12131849; doi:10.1128/spectrum.03186-24)
Supplement: Supplemental material — Supplemental figure legends; Tables S1 and S2. [file spectrum.03186-24-s0005.docx]

**Supplementary Figure legends and Tables**

**Supplementary Figure legends**

**Figure S1. GD Mouse model construction flowchart and verification of adenovirus vector activity and FMT. (A)** Schematic diagram of the GD mouse model construction. **(B, C)** Expression of green fluorescent protein (B) and TSHR protein (C) in 293T cells with different treatments. Ad-TSHR289: 293T cells infected with an adenovirus vector expressing TSHR1-289; Ad-Control: 293T cells infected with an empty adenovirus vector. PBS: 293T cells treated with PBS. **(D)** β-diversity analysis based on 16s rRNA gene sequencing data of gut microbiota from recipient mice before and after FMT and GD patients (donors). n=5. Control: mice intramuscularly injected with adenovirus vector Ad-TSHR289 before transplanted with fecal microbiota from GD patients；GD-FMT: mice intramuscularly injected with adenovirus vector Ad-TSHR289 after transplanted with fecal microbiota from GD patients; GD-donor: GD patients.

**Figure S2. Metabolite detection in** ***B. fragilis* cultures, causal analysis, and animal intervention experiment flowchart and efficacy comparison of inactive and live *B. fragilis*. (A)** Targeted metabolomic analysis of SCFAs in *B. fragilis* cultures, n=4. **(B)** Causal analysis using Structural Equation Modeling (SEM) between *Bacteroides fragilis* levels in the gut and propionate levels in gut and serum. The numbers shown in the figure represent the Path Coefficients, which indicate the strength of the direct causal relationships between variables. **(C)** Schematic diagram of the animal intervention experiment in this study. **(D, E)** Comparison of serum levels of TT4 (D) and TRAb (E) among different groups of mice, n=8. Ad-Control: mice intramuscularly injected with an empty adenovirus vector; GD: mice intramuscularly injected with adenovirus vector Ad-TSHR289 and transplanted with fecal microbiota from GD patients; GD+BF: GD group mice orally supplemented with *B. fragilis*; GD+iBF: GD group mice orally supplemented with inactivated *B. fragilis*; GD+MMI+BF: GD+BF group mice treated with low-dose MMI; GD+MMI+iBF: GD+iBF group mice treated with low-dose MMI. Statistical analysis was performed using One-Way ANOVA with Tukey-HSD test. *: P<0.05; ***: p<0.001; ****: p<0.0001.

**Figure S3. Immunohistochemistry staining of inflammatory cytokines in thyroid tissues of mice.** Representative images of immunohistochemistry staining of inflammatory cytokines IL-1β **(A)**, IL-17 **(B)**, IL-6 **(C)**, and the anti-inflammatory cytokine IL-10 **(D)** in thyroid tissues of mice with different treatments. Ad-Control: mice intramuscularly injected with an empty adenovirus vector; GD: mice intramuscularly injected with adenovirus vector Ad-TSHR289 and transplanted with fecal microbiota from GD patients; GD+MMI: GD group mice treated with low-dose MMI; GD+BF: GD group mice orally supplemented with *B. fragilis*; GD+PA: GD group mice orally supplemented with propionate; GD+MMI+BF: GD+BF group mice treated with low-dose MMI; GD+MMI+PA: GD+PA group mice treated with low-dose MMI.

**Figure S4. Immunofluorescence staining of M1 and M2 macrophages and Treg cells in thyroid tissues of mice.** Representative images of immunofluorescence staining of M1 and M2 macrophages **(A)** and Treg cells **(B)** in thyroid tissues of mice with different treatments. Ad-Control: mice intramuscularly injected with an empty adenovirus vector; GD: mice intramuscularly injected with adenovirus vector Ad-TSHR289 and transplanted with fecal microbiota from GD patients; GD+MMI: GD group mice treated with low-dose MMI; GD+BF: GD group mice orally supplemented with *B. fragilis*; GD+PA: GD group mice orally supplemented with propionic acid; GD+MMI+BF: GD+BF group mice treated with low-dose MMI; GD+MMI+PA: GD+PA group mice treated with low-dose MMI.

**Table S1. Antibodies used in the present study**

| **Antibodies** | **Source** | **Cat. No.** | **Dilution** |
| --- | --- | --- | --- |
| Anti-IL-6 antibody  Anti-IL-1β antibody  Anti-IL-10 antibody  Anti-IL-17 antibody  Anti-iNOS antibody  Anti-FOXP3 antibody  Anti-CD68 antibody  Anti-CD206 antibody  Anti-CD4 antibody  Goat anti-mouse IgG (488-conjugated)  Goat anti-rabbit IgG (488-conjugated) | Abcam  Abcam  Proteintech  Proteintech  Proteintech  Proteintech  Santa Cruz  Abcam  Proteintech  Proteintech  Proteintech | ab290735  ab290735  60269-1-Ig  66148-1-Ig  18985-1-AP  65089-1-Ig  Sc-20060  ab300621  67786-1-Ig  SA00013-1  SA00013-2 | 1:100(IHC)  1:300(IHC)  1:50(IHC)  1:300(IHC)  1:300(IF)  1:300(IF)  1:100(IF)  1:50(IF)  1:500(IF)  1:200(IF)  1:200(IF) |
| Anti-CD4 antibody (FITC-conjugated) | Invitrogen | 11-0042-81 | 1:100(FC) |
| Anti-Foxp3 antibody (PE-conjugated) | Invitrogen | 17-0251-81 | 1:100(FC) |
| Anti-CD25 antibody (APC-conjugated) | Invitrogen | 12-5773-82 | 1:100(FC) |
| Anti-IL-17 antibody (PE-conjugated)  Goat anti-rabbit IgG (PE-conjugated)  Rat anti-mouse IgG (APC-conjugated) | Invitrogen  Proteintech  BioLegend | 12-7177-81  SA00008-2  406609 | 1:100(FC)  1:100 (FC)  1:100(FC) |

**Note:** WB: Western Blot; IHC: Immunohistochemistry; IF: Immunofluorescence; FC: Flow Cytometry

**Table S2. Primers used in the present study**

|  | Primer | Primer sequence (5’–3’) |
| --- | --- | --- |
| Bacteroides fragilis YCH46  *Enterotoxigenic Bacteroides fragilis* (ETBF) *  Total bacteria | Bf YCH-F  Bf YCH-R  ETBF-bft-F  ETBF-bft-R  Univ 27F | GATGAATACTCGCTGTTTG  TTCCACATCATTCCACTG  GGATACATCAGCTGGGTTGTAG  GCGAACTCGGTTTATGCAGTGCGAAC  AGAGTTTGATCMTGGCTCAG |
|  | Univ 1492R | TACGGYTACCTTGTTACGACTT |
| Total bacteria | Univ 337F  Univ 518R | ACTCCTACGGGAGGCAGCAGT  GTATTACCGCGGCTGCTGGCAC |
| Human β-actin  16S rRNA | h-ACTB-F  h-ACTB-R  V3-V4-F  V3-V4-R | GGACTTCGAGCAAGAGATGG  AGGAAGGAAGGCTGGAAGAG  TCGTCGGCAGCGTCAGATGTGTATAAGAGACAGCCTACGGGNGGCWGCAG  GTCTCGTGGGCTCGGAGATGTGTATAAGAGACAGGACTACHVGGGTATCTAATCC |

*: Odamaki T, Sugahara H, Yonezawa S, Yaeshima T, Iwatsuki K, Tanabe S, Tominaga T, Togashi H, Benno Y, Xiao JZ. Effect of the oral intake of yogurt containing Bifidobacterium longum BB536 on the cell numbers of enterotoxigenic Bacteroides fragilis in microbiota. Anaerobe, 2012, 18:14–18. doi: 10.1016/j.anaerobe.2011.11.004.
